# Clinicopathology and Treatment Patterns of Head and Neck Cancers in Ethiopia

**DOI:** 10.1200/GO.22.00073

**Published:** 2022-08-08

**Authors:** Adugna Fekadu, Tara J. Rick, Wondemagegnhu Tigeneh, Eva Johanna Kantelhardt, Luca Incrocci, Ahmedin Jemal

**Affiliations:** ^1^ Department of Radiation Oncology, Addis Ababa University, Addis Ababa, Ethiopia; ^2^ Department of Radiation Oncology, Erasmus MC, Rotterdam, the Netherlands; ^3^ Department of Gynaecology, Martin-Luther-University Halle-Wittenberg, Halle, Germany; ^4^ Surveillance and Health Service Research, American Cancer Society, Atlanta, GA

## Abstract

Head and neck cancers are the third most common cancers treated with radiation in Ethiopia. There is, however, a lack of published data on clinical and pathological characteristics and treatment patterns of head and neck cancers in the country. The objective of the study was to assess clinical and pathological characteristics and treatment patterns of head and neck cancers at Tikur Anbessa Specialized Hospital, which housed the only radiotherapy facility in Ethiopia during the study period.

**MATERIALS AND METHODS:**

A retrospective cross-sectional study design with a simple random sampling of histologically confirmed head and neck cancers treated from 2014 to 2017 with analysis of descriptive data.

**RESULTS:**

Three hundred twenty-one patient charts were analyzed in this study from a total population of 1,377 from the department cancer registry. The male to female ratio was 2:1. The median age was 45 years (interquartile range, 26-59). The most common primary site of head and neck cancers was nasopharynx (128 of 321, 40%), and the major histologic type was squamous cell carcinoma (285 of 321, 89%). Majority of the cases had advanced disease (stage III-IVC, 221 of 251, 88%), but 92% had potentially curable disease (231 of 321). Cobalt radiotherapy was used for 67% of all patients receiving treatment (184 of 273). Induction chemotherapy followed by radiotherapy was frequently used for curative intent patients (75 of 231, 32%). There was long duration between diagnosis and initiation of treatment, with 56% (148 of 264) waiting longer than 3 months.

**CONCLUSION:**

Majority of patients with head and neck cancers seen in Ethiopia presented at advanced stage of disease, received cobalt radiotherapy, and had protracted treatment initiation. These findings underscore the need for additional investments to improve research capacity and increase the availability of high-quality radiotherapy and supportive services to deliver optimal care for patients with head and neck cancer and other cancer patients in the country.

## INTRODUCTION

Head and neck cancers are among the most common cancers worldwide with over 900,000 new cases and 400,000 deaths annually.^1^ They are a heterogeneous grouping of malignancies which have wide geographical differences in epidemiology related to prevalence of risk behaviors and genetic differences among populations. Low- and middle-income countries bear much of the burden and account for 67% of the global cases and 82% of deaths.^2^ In Ethiopia, 3,000 head and neck cancers were estimated to have occurred in 2020, 4% of all cancers.^1^ However, this number is likely underestimated because of barriers to diagnosis and access to care and a lack of nationwide population-based cancer registry.^3^ A previous study reported that head and neck cancers are the third most common type of cancer treated with radiotherapy in the country.^4^

**CONTEXT**

**Key Objective**
What are the treatment patterns of head and neck cancer in Ethiopia, a country with a severe shortage of radiation capacity?
**Knowledge Generated**
The majority of patients with head and neck cancer in Ethiopia presented with advanced stage disease but most still potentially curable. Curative radiation dosing was limited by the limitations of two-dimensional radiation, long wait times, and lack of supportive services.
**Relevance**
There is a need for high-quality three-dimensional radiotherapy with enhanced supportive services to deliver optimal care for patients with head and neck cancer in Ethiopia.

Traditional treatment for curative head and neck cancer includes surgery, radiotherapy, and chemotherapy. However, radiation is currently the mainstay of treatment, and other targeted therapies are increasingly studied and applied. Many factors such as tumor site, stage, functional status, comorbidities, and access to care are taken into account when considering treatment regimens. According to NCCN guidelines, however, patients with localized diseases (stage I-II) are treated with surgery or definitive radiation alone, whereas patients with more advanced disease (stage III-IVb) are treated in a multidisciplinary approach which may include surgery, radiotherapy, and chemotherapy.^5^ For locally recurrent or metastatic disease, palliative therapy and supportive care are typically indicated except in special cases where salvage surgery or radiotherapy may be beneficial.^5^

In Ethiopia, most head and neck cancers are diagnosed at advanced stage, which requires more complicated multimodality curative treatment or palliative strategies.^4^ Until 2020, there was only one cobalt teletherapy machine housed at Tikur Anbessa Specialized Hospital (TASH) in Addis Ababa providing radiotherapy service in the country, a population of over 110 million. The primary aim of this study was to describe clinical and pathological characteristics and treatment patterns of head and neck cancers in Ethiopia.

## MATERIALS AND METHODS

### Study Design and Data Collection

**FIG 1:**
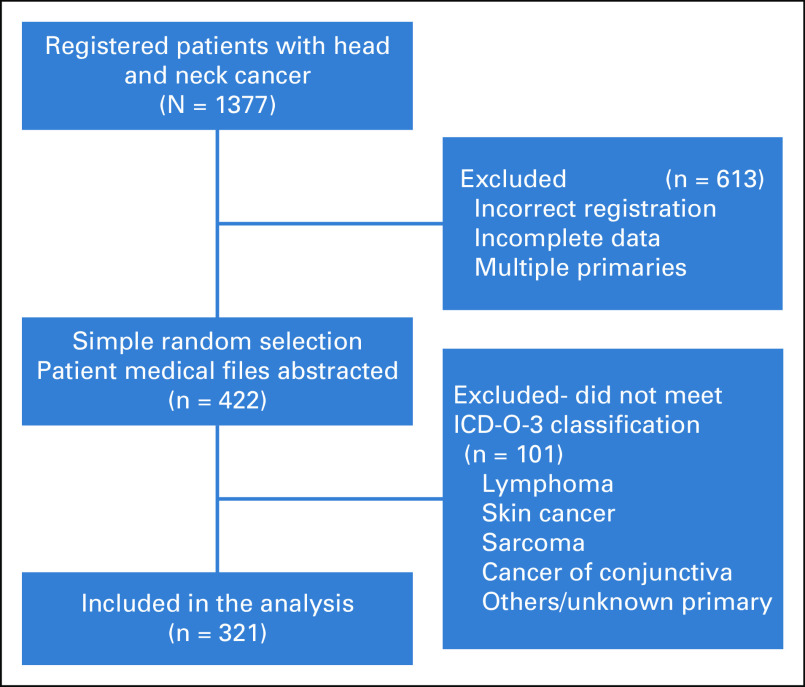
Study flowchart. ICD-O-3, International Classification of Diseases for Oncology, third edition, for Head and Neck Cancer.

This retrospective, cross-sectional study included patients with a diagnosis of head and neck cancer registered at the oncology department of TASH between January 2014 and December 2017. During 2014-2017, 1,377 patients were identified with a new diagnosis of head and neck cancer from the department cancer registry list (Fig 1). The patient medical files were retrieved and reviewed for eligibility, and 613 files were excluded for incorrect registration and for incomplete data (ie, demographics or no histologically verified diagnosis). Next, the remaining 764 medical files were numbered in order of date of diagnosis, and 422 patients were selected with simple random sampling for inclusion in the study. The 422 sample size was used on the basis of sample size calculation using 95% confidence level, 5% margin of error, and 50% population proportion with 10% added for contingency for lost paper charts. Data were then abstracted from the selected files and entered into an SPSS file.

Tumors were classified by primary anatomic site and histology according to the International Classification of Diseases for Oncology, third edition, for head and neck cancer (ICD-O-3). Tumors (n = 93) that did not meet classification criteria were excluded (ie, lymphoma, skin cancer etc). In addition, sarcoma cases (n = 8) were excluded because the diagnosis of Kaposi sarcoma could not be excluded from the information abstracted. The final analysis was based on 321 patients diagnosed with head and neck cancers.

### Study Site, Radiotherapy Resources, and Procedure

TASH is a public referral hospital, and cancer treatment is subsidized by the government with a sliding scale on the basis of income. At the time of the study, it was the only comprehensive cancer center in the country, and patients were referred from all regions of Ethiopia. There was one functional cobalt machine (Theratron Equinox, Best Theratronics, Ottawa, ON, Canada). Two-dimensional treatment planning was used with anatomic landmarks as a planning computed tomography (CT) was not available until a later date. Other radiotherapy resources available included C-arm x-ray simulation, immobilizers, head rest, head and neck masks, and a tattooing device. The quality assurance procedure includes treatment planning by the medical physicists with an oncologist evaluating the plan including the dose distribution and isodose curves for the target.

### Patient Characteristics

Demographic data abstracted from the patient medical file included age, sex, marital status, occupation, region, and whether the patient resided in an urban or rural setting. Other patient characteristics abstracted were comorbidities (HIV/AIDS, diabetes mellitus, hypertension, and cardiovascular disease) and performance status (Eastern Cooperative Oncology Group).

Tumors were classified by primary anatomic and histology according to ICD-O-3 for head and neck cancer. Grade of tumor was recorded for squamous cell carcinoma (well differentiated, moderately differentiated, and poorly differentiated). No information was available on human papillomavirus, Epstein-Barr Virus (EBV), p16 status, or further subcategorization of the squamous cell carcinoma (ie, keratinizing, nonkeratinizing, and basaloid). TNM staging classification (American Joint Committee on Cancer, seventh edition) was abstracted from the chart as recorded by the treating physician. Stage I and II tumors were classified as early stage, and stage III-IVB were classified as locoregionally advanced. Stage IVC had evidence of distant metastatic disease. Routine evaluation to make staging determinations typically consisted of laboratory data such as complete blood count and chemistries and imaging which included chest x-ray, ultrasound, and CT scan. Immunohistochemistry was not available to aid histologic diagnosis. Magnetic resonance imaging scan was available but not routinely used for staging except in selected cases.

### Treatment Modalities and Delay

Treatment modality was abstracted which included no treatment, analgesics only, surgery, chemotherapy, radiotherapy, or combinations of modalities. If chemotherapy was used, the agents and number of cycles were recorded. If radiotherapy was used, the total radiation dose was recorded. The interval between diagnosis and the start of treatment was recorded in months, and patients who initiated treatment after 90 days after diagnosis date were considered delayed.^6^ A single modality approach was defined as treatment with surgery or radiation alone. A multimodality approach was defined as more than one treatment modality such as chemotherapy followed by radiation or concurrent chemoradiotherapy.

### Statistical Analysis

Descriptive statistical analysis was performed using IBM SPSS Statistics version 21 (IBM Corp, Armonk, NY).

### Ethical Considerations

Ethical clearance was obtained from the Medical Faculty Review Board at Addis Ababa University. Our study was executed without individual informed consent because the data were retrospectively obtained from routine care documentation. Confidentiality of the information was maintained with a deidentified database which excluded patient identifiers.

## RESULTS

### Patient Characteristics

**FIG 2:**
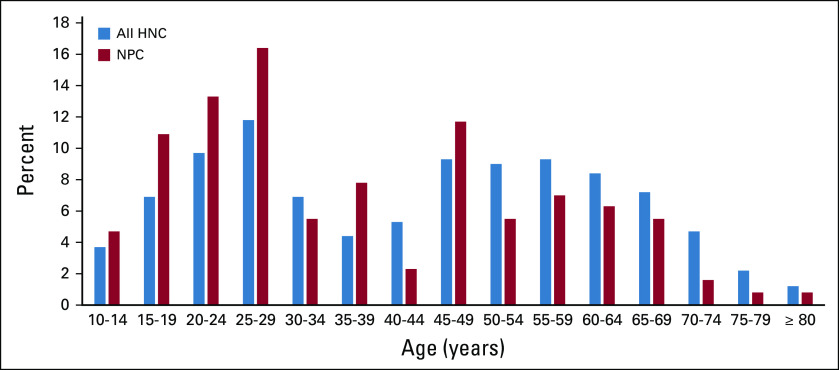
Age distribution of all neck cancers versus nasopharyngeal cancer treated at Tikur Anbessa Specialized Hospital. HNC, head and neck cancer; NPC, nasopharyngeal cancer.

Patient demographic characteristics and risk behaviors are listed in Table 1. The majority of patients were male (67%) and from the regions in closest proximity to the hospital (Oromia/Addis Ababa, 58%). The median age was 45 years for all head and neck cancers and 32 years for nasopharyngeal cancer, age distribution shown in Figure 2. The majority of patients had no reported comorbidity (64%), and 75% had a good performance status (Eastern Cooperative Oncology Group 0 or 1).

**TABLE 1:**
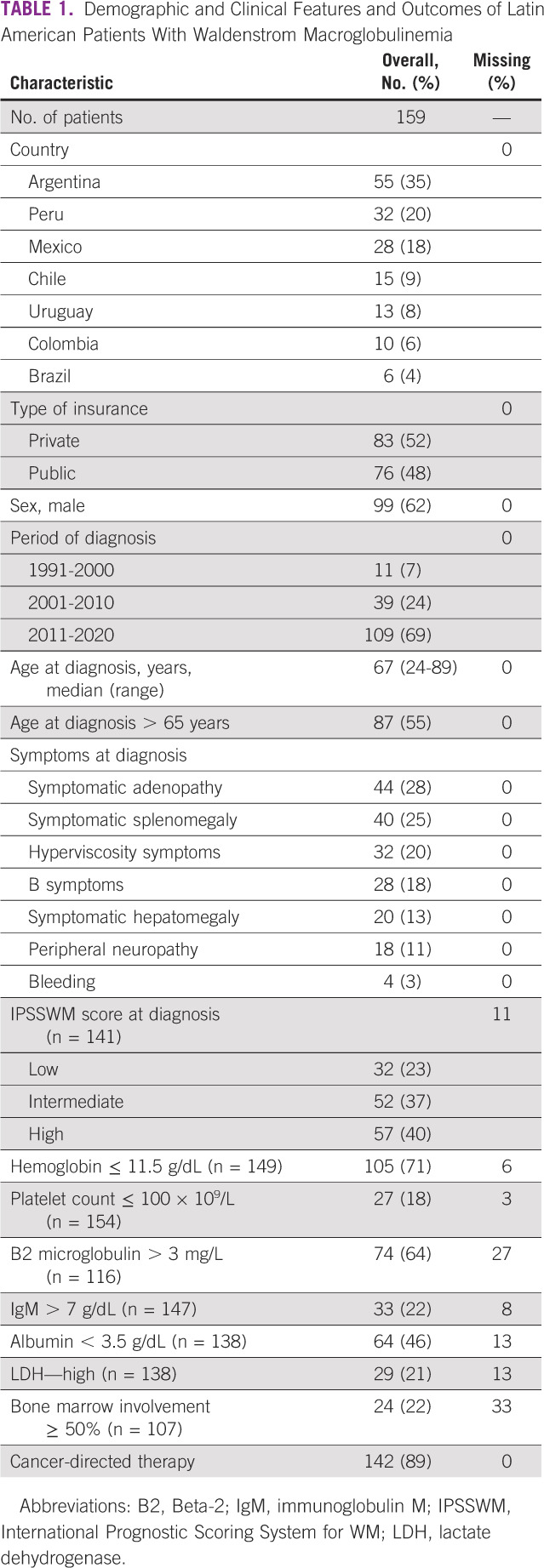
Patient Demographic Characteristics Among Patients Treated for Head and Neck Cancer at Tikur Anbessa Specialized Hospital

### Tumor and Staging

**FIG 3:**
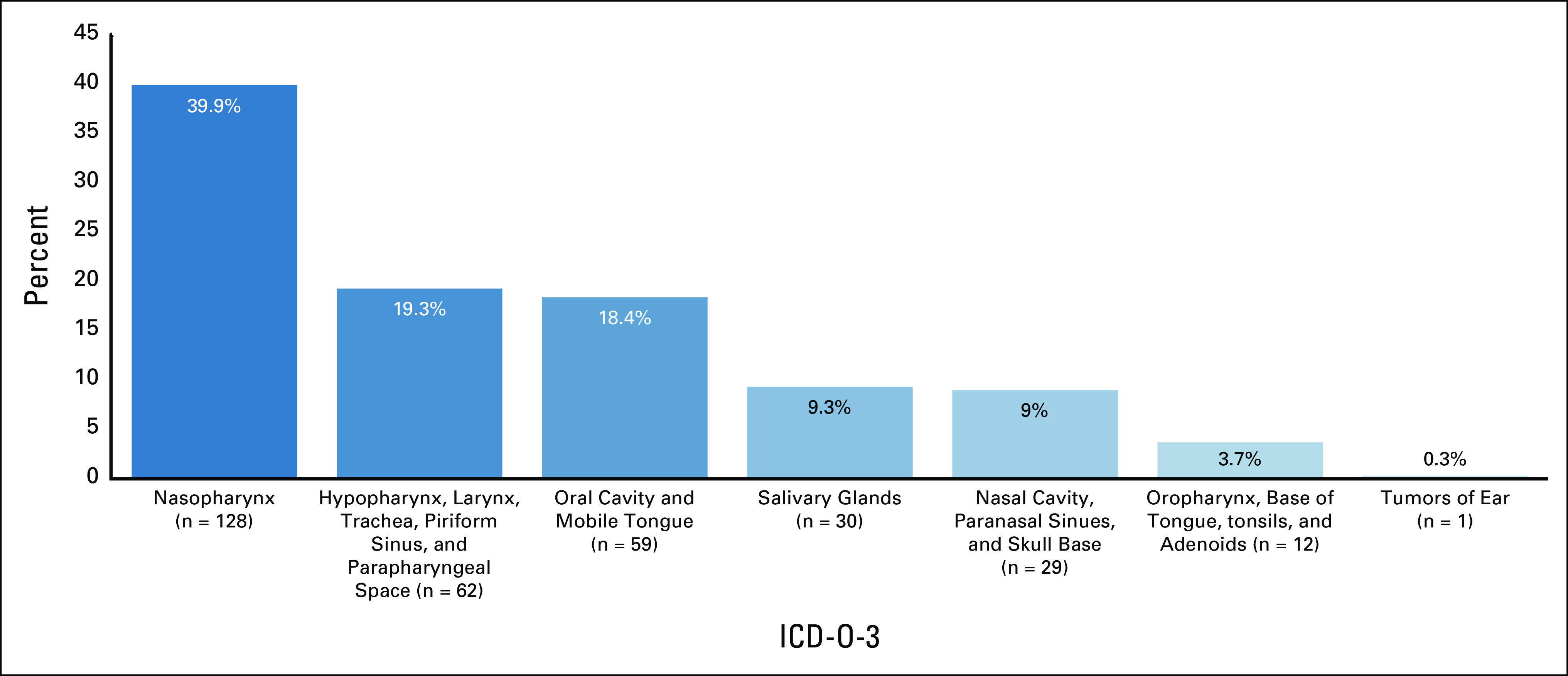
Primary sites of head and neck cancers treated at Tikur Anbessa Specialized Hospital. ICD-O-3, International Classification of Diseases for Oncology, third edition, for head and neck cancer.

The primary sites of head and neck cancers are shown in Figure 3; the most common site being nasopharyngeal cancer (n = 128, 40%) of which most were undifferentiated squamous cell carcinoma (78 of 128, 61%). For all head and neck cancers, squamous cell carcinoma (n = 285, 89%) is the dominant form of head and neck cancers histologic type, with 35% of the tumors well-differentiated and 35% undifferentiated.

**FIG 4:**
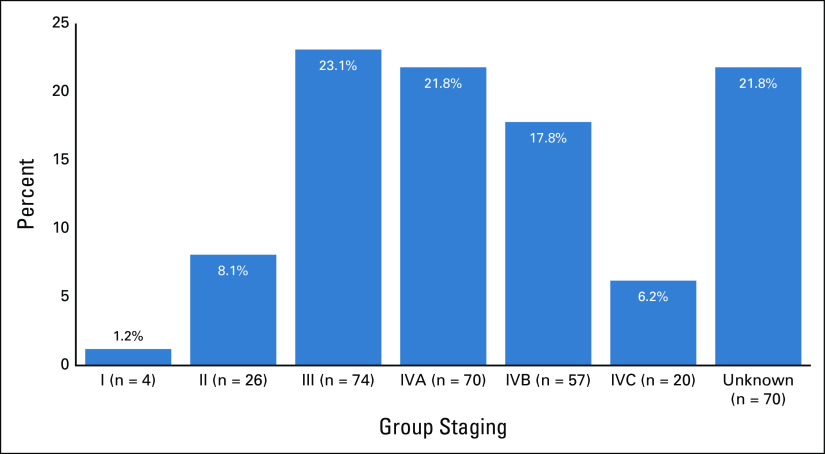
Group staging for head and neck cancers treated at Tikur Anbessa Specialized Hospital (n = 321).

TNM group staging (American Joint Committee on Cancer, seventh edition) is shown in Figure 4. Group staging could not be made in 22% (n = 70) cases because of incomplete T, N, or M records. Therefore, of the 251 cases with staging available, the majority (n = 221, 88%) had locoregionally advanced disease (stage III-IVB) or metastatic disease (stage IVC). However, 92% (231 of 251) were potentially curable (stage I-IVB), and 8% (20 of 251) were incurable (stage IVC).

### Treatment Modalities and Patterns of Care

**FIG 5:**
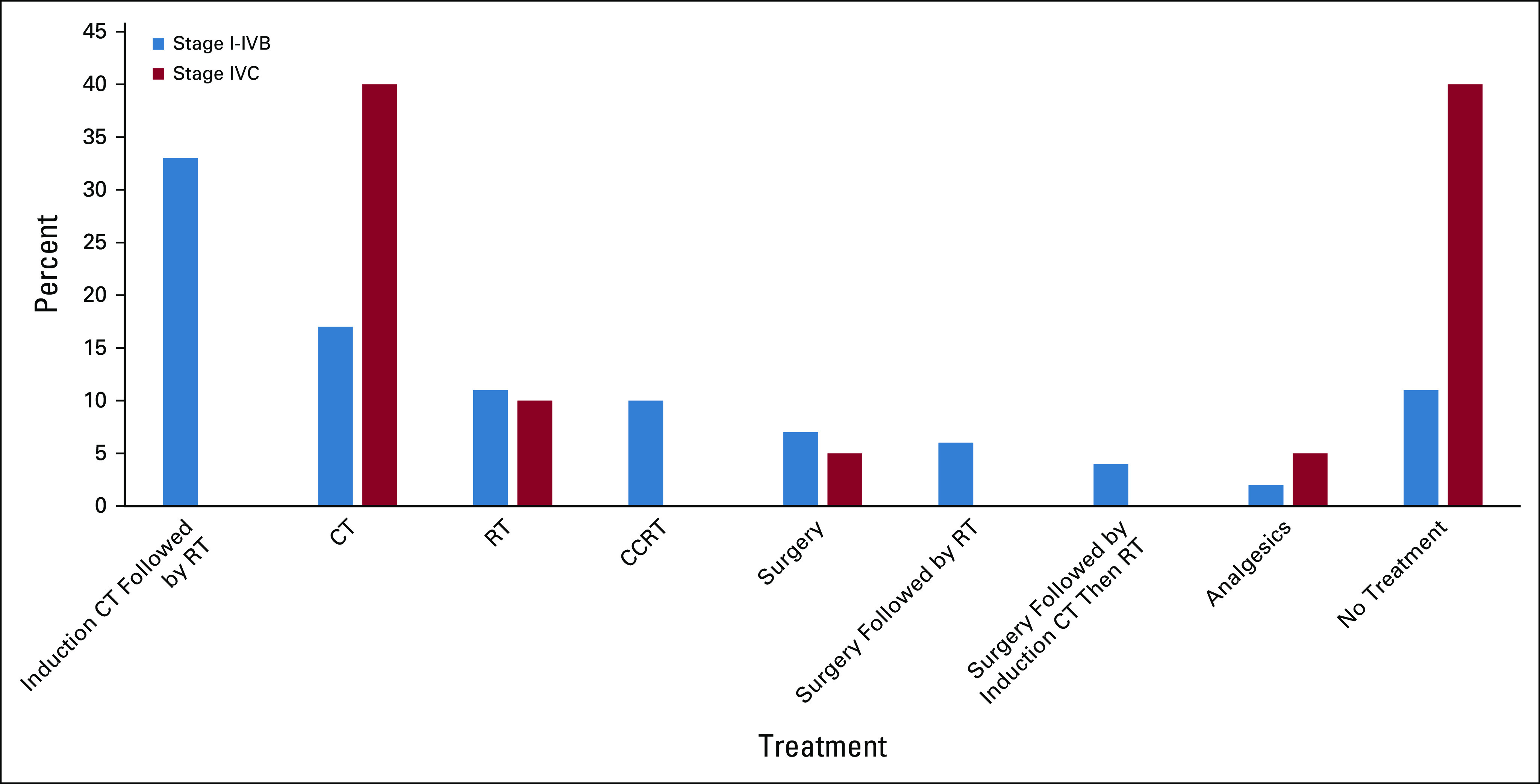
Prescribed treatment regimens for head and neck cancers in Tikur Anbessa Specialized Hospital, stage I-IVB (potentially curable) versus stage IVC (incurable; n = 27). CCRT, concurrent chemoradiation therapy; CT, computed tomography; RT, radiation therapy.

Fifteen percent of the patients (n = 48) were lost to follow-up between diagnosis and being prescribed a treatment regimen; thus, prescribed treatment was recorded for 273 cases. Sixty-seven percent (184 of 273) of the patients were treated with radiation, and radiation dosing was reported for 151 patients. Among the patients with reported radiation dosing, 62% (n = 93) received curative radiation doses over 54 Gy (Fig 5). The most frequently used chemotherapy was cisplatin-containing doublet regimens (158 of 180, 88%) of which a majority received six cycles (76 of 170, 45%).

**FIG 6:**
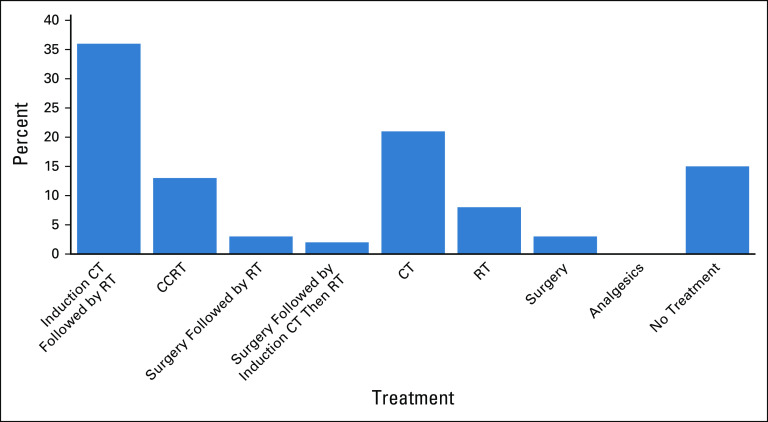
Prescribed treatment regimens for potentially curable (stage I-IVB) nasopharyngeal cancers only in Tikur Anbessa Specialized Hospital (n = 103*). *NOTE: Details of surgery was not collected but based on hospital practice, surgery for pharyngeal cancer likely represents diagnostic surgery. CCRT, concurrent chemoradiation therapy; CT, computed tomography; RT, radiation therapy.

Among 231 patients with potentially curable disease (stage I-IVB), a multimodality approach (n = 121, 52%) was prescribed more frequently than surgery, chemotherapy, or radiation alone (n = 80, 35%); however, only 9.5% of the patients were treated with concurrent chemoradiation (n = 22). Moreover, 11% (n = 25) were not prescribed any form of treatment. Among patients with incurable disease (stage IVC), 55% of patients were treated with palliative, single-modality treatment, 40% received no treatment, and 5% received analgesics alone. The treatment patterns for potentially curable (stage I-IVB) nasopharyngeal cancer are shown in Figure 6; 61% of cases (63 of 103) were treated with radiotherapy alone or in combination with chemotherapy.

### Wait times

**FIG 7:**
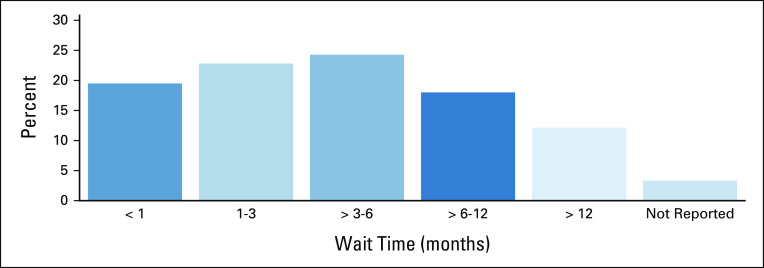
Wait time to start treatment for head and neck cancers at Tikur Anbessa Specialized Hospital (n = 263).

Among the 272 patients who received treatment, 42% (n = 115) waited < 3 months to start treatment, 42% (n = 115) waited between 3-12 months, and 12% (n = 33) waited longer than 12 months. Wait time could not be calculated for 3% (n = 9) of the patients (Fig 7).

## DISCUSSION

To our knowledge, this is the first study to report the clinical and pathological characteristics and treatment patterns of head and neck cancers in Ethiopia. This is based on patients seen in a teaching hospital which houses the only radiotherapy machine in the country to treat head and neck cancers from all parts of the country. It highlights that the majority of head and neck cancers in Ethiopia occur at young age and diagnosed at late stage, that nasopharyngeal carcinoma predominates, and that radiation was frequently used for treatment (curative and palliative), though rarely concurrently, and there was a long wait period to access the one functioning cobalt teletherapy machine in the country.

Our data emphasize a young age of head and neck cancer diagnosis compared with Western countries.^7,8^ This is likely due to nasopharyngeal cancer being the dominant subtype where the age distribution is consistent with intermediate- to high-risk populations with an early peak then decreasing after 50 years in contrast to low-risk populations where risk increases with age.^9^ In high-risk areas, such as in many parts of Asia, nasopharyngeal cancer is associated with EBV, high intake of preserved foods, and early age carcinogen exposure versus the classic head and neck cancer risk factors of tobacco and alcohol use.^10^ It is notable that Ethiopia has low rates of tobacco compared with most regions of the world.^11^ The clinical patterns of nasopharyngeal cancers in Ethiopia is consistent with intermediate-risk endemic areas; however, association with EBV has not been confirmed by analysis of tumor viral EBV DNA.^12^

In this study, the majority of patients had locally advanced head and neck cancer at diagnosis which is observed in countries of high- and low-resource levels alike.^13-16^ Multiple factors influence the timely diagnosis of cancer in Africa such as cultural beliefs, educational level, transportation, and poverty.^17-19^ However, unlike higher resource settings, wait time for initiation of treatment is long in Ethiopia, and during this period, a substantial proportion of patients are lost to follow-up and presumably died without receiving treatment. Long wait time are associated with worse outcomes in head and neck cancers; a study from Taiwan showed worse outcomes after a 40-day delay, and a larger Dutch study showed poorer outcomes after a 90-day delay.^6,20^ It would be logical that long wait times would lead to tumor progression and worse outcomes in our patients; however, longitudinal outcome data were not within the scope of this study.

Radiotherapy is an important organ sparing treatment modality for head and neck cancer; it can be used for definitive treatment, adjuvant therapy to surgery, concurrent with chemotherapy, and for palliation in incurable disease. In Ethiopia, radiotherapy access is limited because of severe capacity issues. The International Agency for Atomic Energy has set an optimal benchmark of between 400 and 500 patients treated per radiotherapy machine per year.^21^ At the time of this study, only one cobalt machine was serving a population over 100 million inhabitants and treating approximately 1,700 patients per year.^22^ This has resulted in long wait times up to and over a year when previously curable tumors may become palliative. There is also much difficulty coordinating the wait lists for chemotherapy and radiotherapy for timely delivery of concurrent chemoradiation therapy which is considered standard of care for locally advanced squamous cell head and neck cancer.^23^ Therefore, the severely under capacity radiotherapy scenario creates barriers for optimal head and neck cancer treatment in Ethiopia.

Radiotherapy dosing depends on multiple factors including type of cancer, staging, and radiotherapy technology. Radiotherapy to the head and neck has the potential to cause considerable acute and chronic complications and morbidity.^24,25^ In recent years, advanced techniques such as intensity-modulated radiotherapy on linear accelerators have been used which maximizes the delivery of radiation to the planned target while minimizing irradiation of normal tissues, resulting in less toxicity and improving outcomes.^26,27^ In this study, definitive radiation doses with cobalt over 54 Gy were considered curative, whereas dosing of 66 Gy or greater is considered standard conventional treatment on a linear accelerator in higher-resource settings.^28-31^ In Ethiopia, radiotherapy was planned and delivered in two dimensions which limit the ability to spare critical structures in the head and neck that cannot tolerate higher doses. This coupled with lack of supportive services such as gastric tube placement and nutrition services limits the maximum dosing of definitive head and neck radiation dosing. For optimal outcomes, a comprehensive head and neck cancer treatment program including dental care, speech/swallowing pathologists, dieticians, and other allied staff are important to manage toxicities to safely deliver curative treatment. However, resources are needed to provide these services. Low- and-middle income countries account for a majority of overall cancer burden around the globe but only have 5% of the worldwide financial resources for cancer care.^32^ Advanced radiotherapy techniques on linear accelerators occurring at a high-volume center with a multidisciplinary approach and access to support services are important for optimal head and neck cancer care.^33^

Treatment patterns of head and neck cancer in Ethiopia are generally similar to those reported in other countries in Africa.^34^ Induction chemotherapy is often used for locally advanced disease as a temporizing measure because of bulky disease and waitlists for radiation. At TASH, typically doublet regimens of cisplatin or carboplatin and either fluorouracil or paclitaxel are used. However, the benefit of induction chemotherapy includes utilization of a triplet regimen (cisplatin, fluorouracil, and a taxane), followed by 7 weeks of chemoradiotherapy.^35-38^ In many centers, feasibility of the triplet regimen as according to the clinical trials and guidelines in resource-rich countries is limited because of several factors including radiation capacity and limited access to granulocyte-stimulating growth factors. Multidisciplinary teams can help guide the rationale implementation of standardized institutional protocols, allowing for evaluating outcomes that can inform future guidelines relevant to the Ethiopian context.

Our study has several limitations. The most important limitation is the large number of participants who were excluded because of poor data quality reported either in the paper chart or incorrectly classified diagnoses in the department registry (according to ICD-O-3 classification) which highlights challenges inherent to retrospective data collection infrastructure. This highlights the importance of investing in high-quality data infrastructure to accurately provide data that are used to make policy decisions. Further prospective data collection should be done to verify our conclusions. Other limitations of this retrospective study design are that it narrows the qualitative descriptors of the data to those previously recorded in the patient file. For example, information on treatment toxicity was not routinely recorded. In addition, staging is predominately anatomical using CT scan for the primary site and regional nodes with chest x-ray and laboratory analysis for distant disease. It is possible that if magnetic resonance imaging and PET/CT were available and routinely used and if missing staging information was included, there could have been further upstaging.^39-41^ Finally, although TASH was the only comprehensive cancer center during the study period, it is possible that it is not a full representative sample of the entire country's incidence or care delivery because it excludes patients who were unable to reach the hospital. It is possible that patients with a poor performance status were unable to travel for treatment.

In conclusion, for improvements in quality of head and neck cancer treatment Ethiopia and data quality, investments need to be made to enhance access to high-quality radiotherapy, particularly intensity-modulated radiotherapy on linear accelerators. In further attempts to improve quality, investment should be made on the quality of data infrastructure as well as availability and expertise of multidisciplinary care and support services.
